# Tomato plants (*Solanum lycopersicum* L.) grown in experimental contaminated soil: Bioconcentration of potentially toxic elements and free radical scavenging evaluation

**DOI:** 10.1371/journal.pone.0237031

**Published:** 2020-08-13

**Authors:** Concetta Piscitelli, Margherita Lavorgna, Rocco De Prisco, Elio Coppola, Eleonora Grilli, Chiara Russo, Marina Isidori

**Affiliations:** 1 Dipartimento di Scienze e Tecnologie Ambientali, Biologiche e Farmaceutiche, Università della Campania “Luigi Vanvitelli”, Caserta, Italy; 2 Consiglio Nazionale delle Ricerche, Istituto di Chimica Biomolecolare, Pozzuoli, Italy; Institute for Biological Research "S. Stanković", University of Belgrade, SERBIA

## Abstract

Tomato is the most widespread vegetable crop in the world. In Italy, tomatoes are mainly cultivated in the South and in the Campania region, precisely in the area called Agro Nocerino-Sarnese. This flatland is affected by an extreme level of environmental degradation, especially related to the Sarno River, where concentrations of Potential Toxic Elements (PTEs) have been found to be higher than the maximum permitted level. The aim of this study was to determine the PTEs uptake by roots and their translocation to the aerial parts of the plants of two cultivars of tomatoes (Pomodoro Giallo and San Marzano Cirio 3). To the purpose, samples of the two cultivars were grown both in pots with experimentally contaminated soil containing: Cr or Cd or Pb at extremely high concentrations and in pots with uncontaminated soils (control). Additionally, the antioxidant properties of the cultivars selected grown on uncontaminated/contaminated soils were assessed. The results showed that Cd was the contaminant that most significantly interfered with the growth of both cultivars of tomato plants, whereas Pb caused lower phenotypical damage. Cd translocation from root to the organs of tomato plants was observed in both cultivars. Specifically, the total amount of Cd found in stems and leaves was higher in the Pomodoro Giallo (254.4 mg/kg dry weight) than in the San Marzano Cirio 3 (165.8 mg/kg dry weight). Cd was the only PTE found in the fruits of both cultivars, with values of 6.1 and 3.9 mg/kg dry weight of Pomodoro Giallo and San Marzano Cirio 3, respectively. The fruits of tomato plants grown in PTEs-contaminated soil showed inhibition or stimulations of the radical scavenging activity compared to the fruits grown in uncontaminated soil. This study highlighted that, despite the relatively high experimental concentrations of PTEs, their translocation to the edible part was comparatively low or absent.

## Introduction

The cultivated tomato, *Solanum lycopersicum* L. endemic to Central and South America, belongs to the family Solanaceae. It is the world’s most highly grown vegetable due to its status as a basic ingredient in a large variety of raw, cooked or processed foods.

Tomato is cultivated in tropical and temperate climates, especially in Mediterranean countries such as Spain and Italy [1]. Nowadays, tomato consumption has increased due to its richness in bioactive compounds such as antioxidant molecules (i.e. carotenoids, flavonoids, vitamins and tocopherols) known for their beneficial effects on human health [2].

However, environmental factors as temperature, and light as well as fertilization of the soil play a key role in the richness of bioactive compounds in different cultivars as well as fruit ripeness [3–5]. Piscitelli et al. [6] focused on San Marzano Cirio 3 and Pomodoro Giallo cultivars while studying the antioxidant and antitumoral properties of nine different cultivars of tomatoes. The authors selected those cultivars for their radical scavenging activity (high polyphenolic content) and their antitumoral properties (content of lycopene and carotene). In Italy, tomatoes are mainly cultivated in the South especially in Agro Nocerino Sarnese (Campania Region) since it is one of the most fertile Italian flatlands for tomato production (31x10^6^ kg of tomatoes in open fields and 44x10^6^ kg tomatoes production and processing in open fields) [7].

This area is unfortunately affected by adverse environmental factors in relation to the Sarno River and its tributaries. The Sarno River is indeed, the most polluted river in Europe due to anthropogenic activity and uncontrolled industrial and urban waste disposal [8, 9]. Many studies have shown that the Sarno river is contaminated by Potentially Toxic Elements (PTEs). Montuori and co-authors [10] demonstrated that this river contributes to the pollution of the Tyrrhenian Sea with metals and polycyclic aromatic hydrocarbons (PHA). In a study conducted by Parrella et al. [9], it is stated that the porewaters of the Sarno basin showed mutagenic/genotoxic effects. Recently, Cicchella and collaborators [11, 12] demonstrated that Sarno stream sediments are contaminated with chromium (Cr) from tanneries and from copper (Cu) released from agricultural activities.

The habit of irrigating crops using river waters has been restricted from the nineties because of the heavily polluted wastewater produced in the tanning industry improperly dumped into the river and its tributaries along a short riverbed (24 Km). Nevertheless, the soil may be contaminated by frequent flooding of the Sarno river, and soil pollution has become a major concern since pollutants persist in this compartment longer than in any other environmental compartment [13]. These pollutants may be absorbed by roots and accumulated at high concentrations in the edible parts of vegetables, thus reaching animals and humans through the trophic chain causing remarkable concern for human health [14–16].

Two studies have focused on the transfer of PTEs from contaminated soil to vegetables grown in the Sarno area [14, 15], indicating the exposure to multiple trace elements with additive, antagonistic, and/or synergistic effects (differently contributing to the potential PTEs translocation).

In contrast with findings from previous studies, the aim of this study was to detect the concentrations of Cr, Cd or Pb occurring in two cultivars grown in pots with experimentally contaminated soil. The PTEs (Cr, Cd or Pb) were selected based on the most occurring heavy metals in the Sarno basin area specifically Cr and Pb occurring in Agro Nocerino Sarnese soil at concentrations higher than the maximum permitted concentrations (152/2006 limits: 150 and 100 mg/kg dry weight, respectively for Cr and Pb) [17]. Cd was considered adequate for its ability of translocation into the fruits of the plants [14]. Thus we assessed the uptake by roots and their translocation to the aerial parts of the plants especially the edible parts. Additionally, the potential modifications in the antioxidant properties of the cultivars selected (Pomodoro Giallo and San Marzano Cirio 3) were assessed. These cultivars were selected on the basis of their high radical scavenging activity, already found in our previous study [6].

## Materials and methods

The experimental plan was conducted in phases proceeding initially with analysis of the physico-chemical properties of the soil to plant the seedlings of two tomato cultivars. Then, the soil was experimentally contaminated in pots with aqueous mono-metal solution of Cd, Cr, Pb (to exclude any environmental pollution, adequate disposal procedures for hazardous residues were followed). On fruit maturation, the roots, stems, leaves and fruits were collected, lyophilized and mineralized to determine the PTE concentrations in the single parts. Only the edible part of the two cultivars was subjected to extraction for free radical activity evaluation.

### Soil analyses

The soil used for PTE contamination was collected in Mondragone 41° 05' 43.2" N—13° 54' 38.5" E, along the Domitio coastline (in Campania Region) at 0–20, 20–40 cm depth (Eutric Arenosols, WRB-SR, 2015). The sample underwent physico-chemical analyses performed on air-dried 2 mm soil (Fine-Earth fraction, f.e.) in accordance with procedures authorized by the Ministero delle Politiche Agricole e Forestali [18]. Soil texture was determined using the pipette method, while soil pH was measured potentiometrically on 1:2.5 soil to water (w/v). Soil electrical conductivity (EC) was measured on 1:5 soil to water (w/v). Organic carbon (OC) was determined according to the Walkley–Black oxidation method and cation exchange capacity (CEC) was measured by barium chloride and triethanolamine (BaCl_2_‐TEA) method at pH 8.2.

### Pot experiments

Seeds of *Solanum lycopersicum* L. cultivars Pomodoro Giallo and San Marzano Cirio 3, were soaked for 15 minutes in tap water and sterilized with sodium hypochlorite (5%) for 15 min. Then, they were washed three times with sterile water and sown in glass beakers with sterile soil. Seeds were exposed to sunlight to germinate at the end of March in a greenhouse. After 30 days the seedlings were transferred into open air to grow in different experimental pots containing 2 kg of the selected soil irrigated with 125 mL of mono-metal solutions for 12 days at increasing percentages equal to 5, 10 and 20% of Cd, Cr and Pb, based on the soil CEC value obtained (cmol(+)/kg f.e.). For each cultivar and for each aqueous solution of PTE at different percentages, three replicates were set up. During cultivation, water was systematically added to soil in pots (maximum 125 mL).

The aim was to simulate growth in an extremely contaminated environment as suggested by Buondonno et al. [19]. The contamination was performed following transplantation to guarantee plant rooting and greater growth potential. For the contamination of the soil, an aqueous solution of cadmium chloride (CdCl_2_) was used as source of Cd; an aqueous solution of chromium hydroxide sulfate [Cr_4_(SO_4_)_5_(OH)_2_] as source of Cr and an aqueous solution of lead nitrate [Pb(NO_3_)_2_] as source of Pb. The different content of SO_4_^2-^ and NO_3_—were equilibrated with addition of KNO_3_ and K_2_SO_4_ to the soil. For each percentage of PTEs (5, 10 and 20%), a respective negative control (PG-NC and PR-NC) was used namely tomato plants (PG and PR) grown in uncontaminated soil, irrigated with water and supplemented with KNO_3_ and K_2_SO_4_ at the percentages of 5, 10 and 20%. The amounts of the compounds added to the soil are reported in Table 1.

**Table 1:** Amounts (expressed in g) of CdCl_2_ (as source of Cd), Cr_4_(SO_4_)_5_(HO)_2_ (as source of Cr) and Pb(NO_3_)_2_ (as source of Pb) added to the experimental soils.

	NC				Cd			Cr			Pb		
	5%	10%		20%	5%	10%	20%	5%	10%	20%	5%	10%	20%
CdCl_2_	-	-	-		1.6	3.1	6.3	-	-	-	-	-	-
Cr_4_(SO_4_)_5_(HO)_2_	-	-	-		-	-	-	1.0	2.1	4.1	-	-	-
Pb(NO_3_)_2_	-	-	-		-	-	-	-	-	-	2.8	5.7	11.3
KNO_3_	1.7	3.4	6.8		1.7	3.4	6.8	1.7	3.4	6.8	-	-	-
K_2_SO_4_	1.3	2.5	4.9		1.3	2.5	4.9	-	-	-	1.3	2.5	4.9

Amounts (expressed in g) of CdCl_2_ (as source of Cd), Cr_4_(SO_4_)_5_(HO)_2_ (as source of Cr) and Pb(NO_3_)_2_ (as source of Pb) added to the experimental soils to obtain Cd, Cr and Pb at increasing levels (5, 10 and 20%), in relation to soil CEC. Amounts of KNO_3_ (as source of NO_3_^-^) and K_2_SO_4_ (as source of SO_4_^2-^) added to uncontaminated soils (Negative Control, NC) and contaminated soils to equilibrate to the concentration of NO_3_^-^ and SO_4_^2-^.

To ensure the radical part as the only experimental source of PTEs, and to exclude the uptake of PTEs from polluted air by absorption through above- ground parts, the tomato plants of the two selected cultivars were grown in an area characterized by the absence of emissions from mobile sources related to vehicle transport and fuels (linear emission), and in absence of emission related to the processes of energy combustion of fuels, industrial processes, discharging substances into the air through point emissions. On fruit maturation, the single parts of the tomato plant (roots, stems, leaves and fruits) were collected. Fruits were cleaned using deionized water to remove dust and soil, while roots were gently shacked to remove the soil attached. The samples derived from the three replicates of each part of the cultivar plants were stored in polyethylene bags for transport to the lab at a constant temperature in dry ice, and stored at -80°C.

### Chemical analysis for PTEs concentrations in tomato fruits, leaves, stems and roots

To detect the concentration of the PTEs [20, 21], in fruits, leaves, stems and roots of PG and PR plants, the samples were lyophilized (- 150 mTorr, - 80°C, 96 h) and then mineralized with hydrogen peroxide (H_2_O_2_) and nitric acid (HNO_3_). The mineralization method consisted in a digestion with hot concentrated acid in closed containers in a diffuse microwave oven (Microwave Digestion System, Start D Model, Milestone) to dissolve metals associated with particles present in colloidal and/or organic forms. Using the mineralization program (Table 2), 2 mL of H_2_O_2_ and 6 mL of HNO_3_ were added to 250 mg of pulverized tomato fruits, leaves, stems and root plant material. The tubes were placed in the microwave for 22 min.

**Table 2:** The mineralization program.

STEP	POWER (W)	TIME (min)	TEMPERATURE (°C)
1	250	1	120
2	0	1	120
3	350	5	160
4	500	5	190
5	800	5	190
6	700	5	190

The mineralization program of Microwave Digestion System.

After the mineralization cycle, the samples were diluted with 50 mL of distilled water, filtered and analyzed by plasma emission spectrometry (ICP-OES) by a ICP-MS (AGILENT, 7500ce).

The concentrations of the selected PTEs found in the negative controls were below the instrumental detection threshold of the ICP-OES analysis (Cr < 0.1 ppm, Pb from 0.1 to 1 ppm, Cd from 0.1 to 1 ppm). The quality control for accuracy and precision of the method was performed with standard reagents for ICP-MS.

Moreover, to assess the environmental impact linked to soil contamination with PTEs, the following parameters were considered-the Bioconcentration Factor (BCF), which is the ratio between the concentration of a PTE detected in plant roots and the concentration of the same PTE detected in the soil; this parameter is an index of the potential absorption of the PTE from the soil to the plant. A BCF value greater than 1 indicates that the element is concentrated in the plant roots [22]; and the Translocation Factor (TF), equal to the ratio between the concentration of a PTE detected in the above-ground parts of the plant and the concentration of the same PTE detected in the roots; this factor refers to the ability of the plant to transfer PTE from roots to stems, leaves, fruits and seeds and may provide information both on the risk of contamination of the edible parts of the plant and on its potential use in phytoremediation techniques.

### Data analysis

The results from three independent experiments were expressed as mean values ± standard error. Statistical analysis was performed by Graphpad Prism5 using *2way-ANOVA*, *Bonferroni*. Differences were considered significant when*p<0.05, **p<0.01. ***p<0.001.

### Free radical scavenging assays

The free-radical scavenging capacity was evaluated by ABTS and DPPH assays on tomato fruit extracts. Tomato fruits were lyophilized and pulverized before extraction. The extraction was then conducted by a solid/liquid ratio of 2:1:1 in which the solvent was a mixture of chloroform, methanol and water, obtaining two fractions: a hydroalcoholic and a lipophilic fraction. The hydroalcoholic fraction was further separated into hydrophilic and methanolic fractions by means of Sep-pak^®^ C18 cartridges. Subsequently, the extracts were filtered and concentrated in a rotary evaporator in vacuum and dried under N_2_. For analysis, the hydrophilic, methanolic and chloroformic extracts obtained were dissolved in water, methanol and chloroform, respectively. The methanolic and chloroform extract stock solutions did not exceed the 5% of methanol and 2% of chloroform.

### ABTS assay

The protocol described by Re et al. and Lavorgna et al. [23, 24] with some modifications was followed. The ABTS radical was generated by adding the potassium persulphate (140 mM) to the ABTS solution (7 mM) for 12–16 h in the dark. Then, the solution was diluted reaching the absorbance of 0.7 ± 0.2 OD at 734 nm. When an antioxidant substance is added to the ABTS solution, the radical is reduced with a consequent discoloration of the reaction mixture.

Subsequently, in the negative control (NC), 1 mL of ABTS solution was added to 100 μL of distilled water and to 100 μL of tomato fruit extracts at different concentrations (100, 200, 500 and 1000 mg/L). The distilled water was used for the blank and Trolox was used as a standard antioxidant. After 6 min, the absorbance of solution was measured at 734 nm. The percentage of absorbance decrease was calculated according to the following equation (1):

$$Inhibition\;(\%)=\frac{{OD}_{734}radical-{OD}_{734}sample}{{OD}_{734}radical}.100$$

### DPPH assay

DPPH radical scavenging activity of the tomato fruit extracts was performed following the protocol described by Brand-Williamset al. and Pacifico et al. [25, 26]. 940 μL of DPPH (101.43 μM) was added to 60 μL of distilled water for negative control (NC) or to 60 μL of tomato fruit extract at different concentrations (100, 200, 500 and 1000 mg/L). The methanol solution was used for the blank, and Trolox was used as a standard antioxidant. The absorbance of DPPH solution was measured at 517 nm after 30 min. The percentage of absorbance decrease was calculated according to the equation (2):

$$Inhibition\;(\%)=\frac{{OD}_{517}radical-{OD}_{517}sample}{{OD}_{517}radical}.100$$

### Data analysis

The outcomes from three independent experiments were expressed as EC50 (the median effective concentration of sample able to reduce the initial absorbance of the ABTS and DPPH radical) values. The EC50 values were obtained by Graphpad Prism5 analysis by non-linear regression (log agonist vs. normalized response-variable slope) with 95% confidence range. The results were analyzed by the *unpaired t-test*. Differences were considered as significant when *p* values were *p<0.05 and **p<0.01.

## Results

The soil showed a sandy texture and the results of physicochemical analyses revealed a moderately alkaline pH (8.0, in H2O). The electrical conductivity was 0.48 dS/m, the OC was 17.2 g/kg while CEC value was 17.1 (cmol(+)/kg).

At harvest time, Pomodoro Giallo and San Marzano Cirio 3 plants, grown in experimental contaminated soils, showed clear phenotypical alterations (S1 and S2 Figs). These changes were especially evident for the biomass, expressed as dry weight of the plants. The dry weight of PG plants with 20% of Cd, Cr and Pb (based on the soil CEC value obtained, cmol_(+)_/kg f.e.) was 85%, 53% and 32%, respectively, less than the dry weight of the negative control (136.5g). The dry weight of PR plants with 20% of Cd, Cr and Pb was 82%, 48% and 23%, respectively, less than the dry weight of the respective negative control (102.7g). Among all PTEs, Cd was the main contaminant able to interfere with the growth of the plants of both tomato cultivars. Specifically, at Cd 20%, Pomodoro Giallo plants did not produce fruits, while the San Marzano Cirio 3 plants produced few fruits. As regards Cr, most of the plants showed poor vegetation and scarce fruit production. Finally, among the PTEs tested, Pb was the contaminant responsible for less phenotypical damage to the plants. Moreover, between the two cultivars, Pomodoro Giallo appeared more sensitive to the contamination of the PTEs.

### ICP-OES analysis

The concentrations (mg/kg dry weight) at increasing percentages of Cd, Cr and Pb are reported in Table 3.

**Table 3:** Concentrations (mg/Kg dry weight) of Cr, Cd and Pb, detected by ICP-OES analysis, in bulk soil, roots, stems, leaves and fruits of PG and PR.

		Bulk soil		Roots		Stems		Leaves		Fruits	
		PG	PR	PG	PR	PG	PR	PG	PR	PG	PR
**Cr**	**5%**	22.6±7.5	9.9±1.0	**15.0±1.2**	8.9±0.6*	0	0	0	0	0	0.4±0.2
	**10%**	65.9±5.2	80.1±17.4	**17.3±1.5**	0.8±0.1***	0.2±0.1	0.5±0.1	1.7±1.2	0.8±0.1	0.5±0.1	0.4±0.1
	**20%**	119.3±38.6	53.1±5.7	**51.9±2.0**	27.9±1.9***	0.4±0.3	0	0	0.2±0.1	0	0
**Cd**	**5%**	137.4±6.0	159.0±16.0	**45.2±3.4**	143.9±13.6***	26.4±0.7	32.2±2.3	32.1±1.8	41.0±0.7	**2.1±0.1**	3.8±0.1***
	**10%**	**426.2±64.2**	151.3±8.0**	**114.9±11.3**	82.1±3.0*	**54.2±2.1**	26.4±3.8***	**94.5±0.6**	20.7±0.3***	**6.1±0.3**	3.9±0.2***
	**20%**	**879.0±53.2**	614.5±13.9**	**59.7±3.7**	103.2±9.2*	**181.6±4.6**	64.9±3.0***	**72.8±6.7**	100.9±1.4***	No fruits	0.7±0.2
**Pb**	**5%**	903.6±56.8	562.8±11.4	338.3±18.2	259.0±40.0	**9.3±0.3**	4.4±0.4***	0	0	0	0
	**10%**	1545.4±136.5	1580.6±63.0	929.9±46.5	751.3±131.1	10.8±0.5	11.9±0.8	**2.2±0.1**	0.9±0.1***	0	0
	**20%**	2657.1±104.8	2653.0±100.7	**941.8±87.7**	1572.1±117.7***	**19.3±1.2**	10.5±0.1***	**2.7±0.2**	0.7±0.1***	0	0

Concentrations (mg/Kg dry weight) of Cr, Cd and Pb, detected by ICP-OES analysis, in bulk soil, roots, stems, leaves and fruits of PG and PR. Results are reported as mean values (3 independent experiments) ± Standard Error. Significant differences between PG and PR for each part and level% CEC of Cr, Cd and Pb were analysed by 2way-ANOVA, Bonferroni for *p<0.05, **p<0.01, ***p<0.001.

Considering the different dislocation of the PTEs along the plant organs, it is noteworthy that Pb and Cr were found in roots, at the highest tested percentages (20%), in the order of hundreds/thousands and tens of mg/kg dry weight respectively, decreasing to values close to few mg/kg in stems and leaves. Interestingly, Pb was found in roots at 5% at 338.3 and 259 mg/kg in PG and PR, respectively. Furthermore, an increasing PTE%-recovering trend dependent on the quantity of Pb added in the soil was observed, reaching 20%, values equal to 941.8 (PG) and 1572.1 (PR) mg/kg. At the same level (20%) translocation of Cd from root to the organs of tomato plants was observed in both tomato cultivars. Significant differences between PG and PR for each part were observed for *p<0.05, **p<0.01, *** p<0.001. Specifically, the total amount of Cd found in stems and leaves was higher in Pomodoro Giallo (254.4 mg/kg) compared to San Marzano Cirio 3 (165.8 mg/kg).

Table 3 illustrates evident differences in the PTE concentrations of the fruits. Surprisingly, Cd (10%) was the only PTE found in the edible parts of Pomodoro Giallo at 6.1 mg/kg dry weight (about 0.5 mg/kg wet weight) and San Marzano Cirio 3 at 3.9 mg/kg dry weight (about 0.3 mg/ kg wet weight). These concentrations were higher than those recommended by the Commission Regulation (EC) No.629/2008 that sets maximum concentrations for Cd in food stuffs at 0.05 mg/kg wet weight. Concurrently, the Italian legislative decree 152/2006 sets the limit for Cd in the soil at 2 mg/kg dry weight, a significant lower concentration compared to the quantities adopted herein to irrigate the soil, equal to 2 g.

In addition, in the present study, BCF and TF values were calculated in relation to soil Cation-exchange Capacity (Table 4). The PTEs analyzed were not able to concentrate in tomato plant roots (BCF< 1) and only Cd showed the ability to move from roots into the above- ground parts of the tomato cultivars (TF = 4.262 in PG and 1.613 in PR, at 20%). Pomodoro Giallo plants showed a higher translocation potential of Cd from the roots to the above-ground parts of plant than San Marzano Cirio 3 plants, with TF values higher than 1 already at the lowest percentage of Cd used for soil contamination.

**Table 4:** BCF and TF values.

		Pomodoro Giallo		San Marzano Cirio 3	
PTE		BCF	TF	BCF	TF
Cd	5%	0.329	1.340	0.905	0.535
	10%	0.270	1.347	0.543	0.621
	20%	0.068	4.262	0.168	1.613
Cr	5%	0.663	0	0.903	0
	10%	0.264	0.135	0.010	0.141
	20%	0.435	0.008	0.526	0.001
Pb	5%	0.374	0.027	0.460	0.015
	10%	0.602	0.014	0.475	0.017
	20%	0.354	0.023	0.593	0.007

BCF values (bioconcentration factor) and TF values (translocation factor) of Cd, Cr and Pb at different percentages (5, 10 and 20%) in relation to soil Cation-exchange Capacity, in the two tomato cultivars, Pomodoro Giallo and San Marzano Cirio 3.

### Free radical scavenging evaluation

Measurements of tomato free radical scavenging activity were carried out on the fruits of the tomato plants irrigated at PTE contamination levels of 10% of CEC, the highest level of PTEs resulting in plant fructification.

The results, expressed as EC50 values, are reported in Tables 5 and 6 for Pomodoro Giallo and San Marzano Cirio 3, respectively. It was observed that both tomato fruit extracts coming from uncontaminated and contaminated soils showed antioxidant activity. Among the extracts, the hydrophilic and methanolic extracts showed a higher free-radical scavenging capacity in both ABTS and DPPH assays, compared to chloroformic extracts which presented EC50 values generally higher than the other fractions. In particular, for the DPPH assay, the hydrophilic extracts of PG tomatoes grown on soil contaminated with Cr, showed a significant EC50 value (52.9 mg/L, p<0.01) compared to the negative control EC50 (339.4 mg/L), demonstrating the highest antiradical activity followed by the methanolic extracts (EC50 = 99.7 mg/L, p<0.01) and by the chloroformic extracts (EC50 = 304.1 mg/L, p<0.05) of plants grown in presence of Cd. Conversely, significant increases of EC50 values were observed in both tests, highlighting a reduction of the antiradical scavenging capacity for some fractions of the cultivars exposed to the selected PTEs, especially for PR in the methanolic extracts.

**Table 5:** Free-radical scavenging capacity evaluated by ABTS and DPPH assays of tomato extracts of Pomodoro Giallo.

		Pomodoro Giallo (PG)	
		ABTS assay	DPPH assay
Hydrophilic extracts	NC	123.7 (106.0–144.2)	339.4 (243.1–473.8)
	Cd	184.9 (154.6–221.2)	383.7 (318.6–462.0)
	Cr	127.3 (106.8–151.7)	52.9** (39.1–71.8)
	Pb	160.4 (120.4–213.8)	883.3* (754.4–918.0)
Methanolic extracts	NC	122.5 (109.3–137.4)	196.6 (177.2–218.1)
	Cd	148.8 (125.8–176.0)	99.7** (90.5–110.0)
	Cr	107.5 (66.3–174.2)	538.9* (341.6–902.2)
	Pb	112.3 (80.6–156.5)	206.9 (124.5–343.8)
Chloroformic extracts	NC	814.9 (546.5–1015.0)	976.1 (722.0–1020.0)
	Cd	751.6 (491.1–1050.0)	304.1* (189.2–488.5)
	Cr	754.4 (585.4–972.3)	901.7 (760.8–1059)
	Pb	751.1 (497.9–103.3)	945.3 (728.3–1027.0)

Free-radical scavenging capacity evaluated by ABTS and DPPH assays and expressed as EC50 (mg/L, with 95% confidence limits in brackets) of tomato extracts of Pomodoro Giallo grown in uncontaminated (NC) and contaminated (Cd, Cr, Pb) soils. A significant difference from NC was determined with Unpaired t-test*p<0.05, **p<0.01.

**Table 6:** Free-radical scavenging capacity evaluated by ABTS and DPPH assays of tomato extracts of San Marzano Cirio 3.

		San Marzano Cirio 3 (PR)	
		ABTS assay	DPPH assay
Hydrophilic extracts	NC	116.3 (101.8–132.8)	246.9 (218.4–279.2)
	Cd	118.4 (105.2–133.4)	393.6 (290.3–533.7)
	Cr	143.3 (109.3–187.9)	568.8* (358.4–752.0)
	Pb	143.0 (113.9–179.5)	699.9* (538.0–910.6)
Methanolic extracts	NC	113.8 (98.13–131.9)	102.1 (68.86–151.3)
	Cd	455.1* (373.0–555.3)	159.3 (146.1–173.7)
	Cr	222.3* (186.3–265.4)	121.4 (105.0–140.3)
	Pb	233.8* (239.8–296.8)	99.9 (83.9–118.8)
Chloroformic extracts	NC	568.3 (380.3–849.0)	697.3 (396.2–1027.0)
	Cd	809.9 (734.4–893.0)	294.6 (168.6–514.9)
	Cr	649.7 (517.7–810.3)	759.5 (571.3–1010.0)
	Pb	763.2 (597.8–974.2)	910.8 (763.9–1086.0)

Free-radical scavenging capacity evaluated by ABTS and DPPH assays and expressed as EC50 (mg/L, with 95% confidence limits in brackets) of tomato extracts of San Marzano Cirio 3 grown in uncontaminated (NC) and contaminated (Cd, Cr, Pb) soils. A significant difference from NC was determined with *Unpaired t-test* *p<0.05, **p<0.01.

## Discussion

Results differed according to the PTEs under analysis; each one showing peculiarities noteworthy of further discussion.

Cd was able to interfere with the growth of both examined tomato cultivar plants. In particular, at 20%, both Pomodoro Giallo and San Marzano Cirio 3 plants showed scarce development, Pomodoro Giallo plants did not produce fruit, while the San Marzano Cirio 3 plants had a low production. These results may be due to the high potential of Cd to be absorbed by roots and accumulated in all the plant organs and tissues, especially in metabolically less active parts of leaves [27]. The lack or the reduced quantity of fruit observed in the plants exposed to the highest concentration of Cd and Cr was most likely caused by the effect of PTEs on enzymes or other metabolites or by their ability to generate reactive oxygen species which may yield oxidative stress [28]. In addition, Cr is able to easily modify its oxidation state causing the formation of free reactive oxygen species (ROS) [29, 30] which may result in: lipid peroxidation, damage of the chloroplast ultrastructure, reduction of photosynthetic activity or of chlorophyll biosynthesis, reduction of growth and alteration of water balance [31]. Conversely, some studies have reported that Cr, in the form of Cr (III), is able to increase plant growth at low concentrations [32, 33]. Furthermore, this element may produce positive effects on plant growth influencing the production of cytokinins or other growth hormones [34]. As Cr may have a different impact on plant growth, similarly, plants treated with Cd may or may not show signs of toxicity [27]. To improve and/or stimulate the growth and development of plants in soils contaminated by Cd, Rao and co-authors [35] recently suggested ultrasonic seed treatment which increases cadmium tolerance. Finally, among the PTEs tested, Pb was the contaminant that caused less phenotypical damage to the plants. Hu et al. [36] reported that Pb showed low absorption potential through roots. Differently, Ashraf and Tang [37] showed that Pb toxicity severely reduced yield and quality- related attributes and plant biomass in two aromatic rice cultivars. In addition to phenotypical responses under stressful environment with PTEs, quantitative analyses were performed on the mineralized samples of the exposed plants. As reported in the results, differences in concentrations of the single PTEs were found in the roots, and in the edible parts of the two cultivars. At the highest percentage of PTE tested, Pb was found mainly in the roots, in the order of hundred/thousands of mg/kg dry weight (bulk soil concentration of about 2650 mg/kg dry weight), and its transport to the aerial parts was extremely limited and was absent in the fruit. Cr showed a different trend compared to Pb with a concentration in the order of tens of mg/kg dry weight in the roots (bulk soil concentration ranging from 53.1 (PR) to 119.3 (PG) mg/kg dry weight), with irrelevant concentrations in stems, leaves and fruit. On the contrary, Cd had a higher ability to transport from roots to aerial parts compared to Cr and Pb. However, it must be considered that the experimental design of this study was characterized by a strong contamination of the soil in order to generate perceptible changes in the plant PTE intake capacity and in its metabolism. Moreover, the BCF values revealed that none of the PTEs was able to bio-concentrate in tomato plant roots, showing BCF values lower than 1. However, the TF values strongly suggest that among the three PTEs, only Cd showed partial ability to translocate into the above- ground parts of the plants. Additionally, the Pomodoro Giallo plants showed a stronger ability of translocation of Cd compared to San Marzano Cirio 3 plants, with TF values higher than 1 even at the lowest percentage (5%) of Cd. These results are confirmed by Trebolazabala and co-authors [38], who assessed the concentration of some potentially toxic elements, including Cd, Pb and Cr, in soils and in the different parts of 11 tomato cultivars, grown in Biscay, in Northern Spain. These authors observed that the concentrations of such PTEs were significantly higher in the soil than in the plants and that PTEs were accumulated in roots and leaves but not in the fruit. Similarly, in the study of Opeolu et al. [39], Pb accumulated in the roots, but not in the fruit. On the contrary, in a recent study, Magaji and collaborators [40] detected heavy metals (Cd, Cu, Pb and Zn in mg/kg) in vegetables obtained from Kembu farms and observed that only Pb exceeded the permissible limit of 0.3 mg/kg in tomatoes. Finally, in accordance with the results of the present study, Gharaibeh and collaborators [41] showed that tomato plants, grown in the presence of Cd, had a higher concentration in the roots compared to the fruit. In another study, Raptis and co-authors [42] showed that the uptake of Cr in lettuce shoots and roots increased in line with concentration of Cr in irrigation water. However, in the presence of high PTE concentration in the soil, most plants tend to limit the intake in the aerial parts and store toxic elements in the roots as they are not required for vegetal growth [15]. In addition, tomatoes show the potential to produce a higher content of secondary metabolites to protect themselves from toxic effects resulting from exposure to PTEs [14].

The results obtained from the PTEs at 10% of CEC of the soil suggest that the antioxidant activities of the tomato fruits can be limited or stimulated. The exposure to Cd at this level determined an increase of the antiradical scavenging activity (DPPH assay), in the methanolic and chloroformic extracts of PG fruits. In PG plants grown in uncontaminated soil, fruits were characterized by methanolic extract rich in polyphenols (474 mmol quercitin equivalents/kg of fresh products), and hydroxycinnamic acids such as caffeic acid and by the chloroformic extract rich in carotene as reported by Piscitelli et al. [6]. The exposure to Cr at the same level of Cd (10% of CEC) determined a significant increase of the antiradical scavenging activity (DPPH assay) in the hydrophilic extract of PG which is rich in polar phenolic compounds such as rutin and chlorogenic acid, hydro-soluble vitamins such as ascorbic acid. Differently from PG, PR did not show increase in radical scavenging properties with significant reduction in ABTS scavenging activity of methanolic extracts for each PTE. Therefore, it is most likely that the variety of tomato affects the tolerance to Cd, Cr and Pb. For example, some studies report high levels of antioxidant-producing enzymes (superoxide dismutase, ascorbate peroxidase and catalase) of tomatoes contaminated by heavy metals [43–45], contributing to the increment of biological properties in tomato fruits. These biochemical changes which increase the antiradical scavenging performance in the case of PG may be due to a possible hormetic effect, a stimulatory response to a certain level of soil contamination and inhibitory effect at higher levels. Indeed, in the present study, for higher levels of PTEs (20% of CEC) the plants did not bear fruits or died. The effects observed in fruit of the plants grown at the highest level of Cr and Cd were likely caused by their potential ability to generate oxygen species which may cause lipid peroxidation, reduction of photosynthetic activity and of chlorophyll biosynthesis, reduction of growth or alteration of water balance [31]. This oxidative stress may decrease adaptive responses of plants to metal ion homeostasis and redox balance precluding the switch in growth/defence equilibrium.

## Conclusions

Differently from Cr and Pb which did not confirm translocation in the edible part of the plants, Cd was able to reach the fruits albeit at very low concentrations, significantly lower than those established by the regulation for fresh fruits. Despite Cd translocation, phenotypical effects were not observed up to a contamination level of 10% of CEC while significant differences in the antiradical properties were found for each PTE and for each cultivar. Types, concentrations and activities of PTEs modulated antiradical mechanisms with stimulatory or inhibitory responses, which most likely depended on the content of secondary metabolites produced by tomato plants in an effort of self-protection against the exposure to such elements. This study highlighted that, despite the experimental higher concentrations of PTEs compared to those occurring in the Sarno basin, strongly contaminated by the PTEs herein analyzed, the translocation of the elements to the edible part was rather low or absent. The potential health risk due to the vegetable consumption of crops grown in soils contaminated at very lower concentrations, in any case it is not enhanced also because of the ability of the tomato plants to reduce the PTEs transfer from the soil to the fruits. However, further studies from genetic-molecular to physiological and ecological levels are required for a better understanding of defensive/adaptive response mechanisms resulting from tomato plants exposed to toxic elements. Furthermore, the contribution of vegetable species consumption may be further developed to acknowledge potential health risks for residents in contaminated areas especially in a vulnerable population, such as children.

## Supporting information

S1 Fig Pomodoro Giallo plants grown in experimental contaminated soils. Pomodoro Giallo plants, grown in experimental soils contaminated with Cd, Cr and Pb at increasing levels (5, 10 and 20%) in relation to soil CEC values. From the top to the bottom, untreated tomato plants (NC = Negative Control) and plants treated with Cd, Cr and Pb.
(DOCX)

S2 Fig San Marzano Cirio 3 plants grown in experimental contaminated soils. San Marzano Cirio 3 plants, grown in experimental soils contaminated with Cd, Cr and Pb at increasing levels (5, 10 and 20%) in relation to soil CEC values. From the top to the bottom, untreated tomato plants (CN = Negative Control) and plants treated with Cd, Cr and Pb.
(DOCX)
