# Supplementary material for: Tomato plants (Solanum lycopersicum L.) grown in experimental contaminated soil: Bioconcentration of potentially toxic elements and free radical scavenging evaluation
Source: PLoS One. 2020 Aug 13;15(8):e0237031. doi: 10.1371/journal.pone.0237031 (PMC7425901; doi:10.1371/journal.pone.0237031)

**Fig. S2.** **San Marzano Cirio 3 plants, grown in experimental contaminated soils**. San Marzano Cirio 3 plants, grown in experimental soils contaminated with Cd, Cr and Pb at increasing levels (5, 10 and 20%) in relation to soil CEC values. From the top to the bottom, untreated tomato plants (CN = Negative Control) and plants treated with Cd, Cr and Pb.
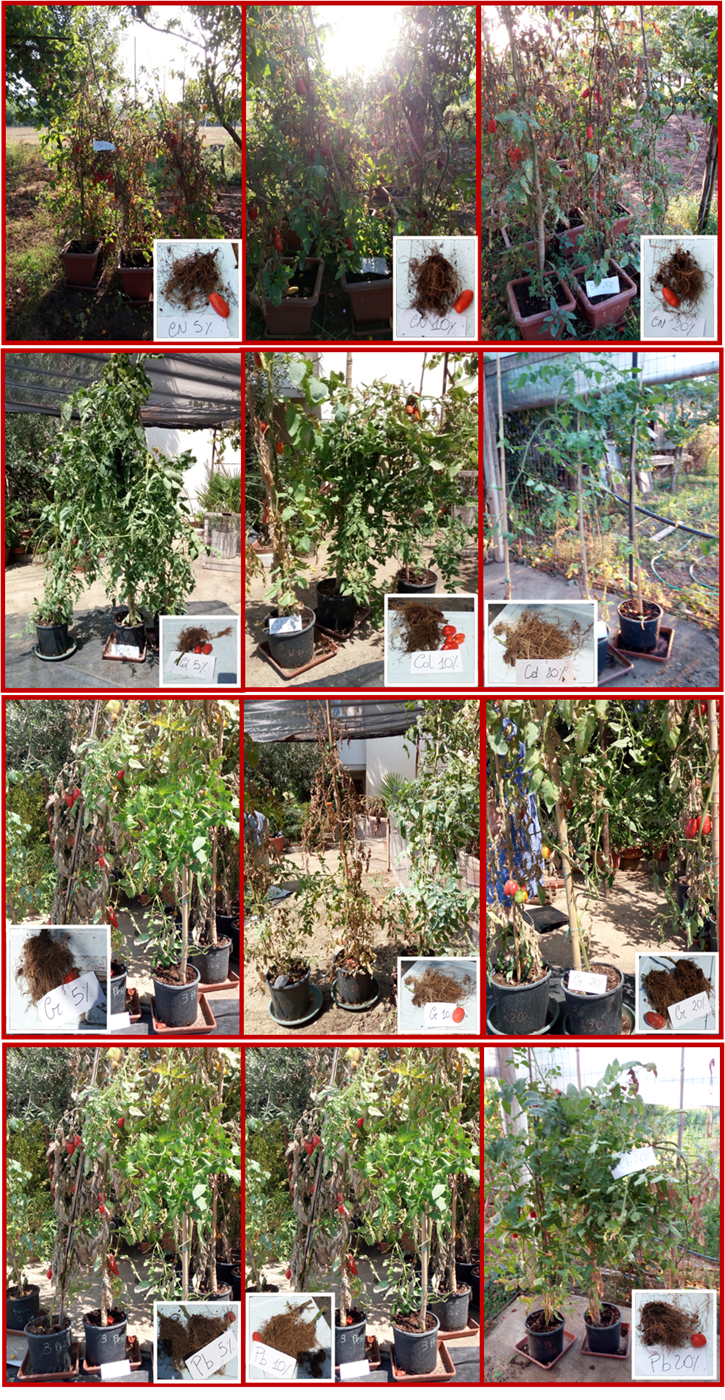

Supplement: S2 Fig — San Marzano Cirio 3 plants, grown in experimental soils contaminated with Cd, Cr and Pb at increasing levels (5, 10 and 20%) in relation to soil CEC values. From the top to the bottom, untreated tomato plants (CN = Negative Control) and plants treated with Cd, Cr and Pb. (DOCX) [file pone.0237031.s002.docx]
